# Enhancement of Ammonium Oxidation at Microoxic Bioanodes

**DOI:** 10.1021/acs.est.3c02227

**Published:** 2023-07-27

**Authors:** Xiaofang Yan, Dandan Liu, Johannes B. M. Klok, Sanne M. de Smit, Cees J. N. Buisman, Annemiek ter Heijne

**Affiliations:** † Environmental Technology, Wageningen University & Research, P.O. Box 17, 6700 AA Wageningen, The Netherlands; ‡ Paqell B.V., Reactorweg 301, 3542 AD Utrecht, The Netherlands; § Wetsus, European Centre of Excellence for Sustainable Water Technology, Oostergoweg 9, 8911 MA Leeuwarden, The Netherlands

**Keywords:** Ammonium, bioanode, oxygen, ammonia-oxidizing microorganisms, electro-anammox

## Abstract

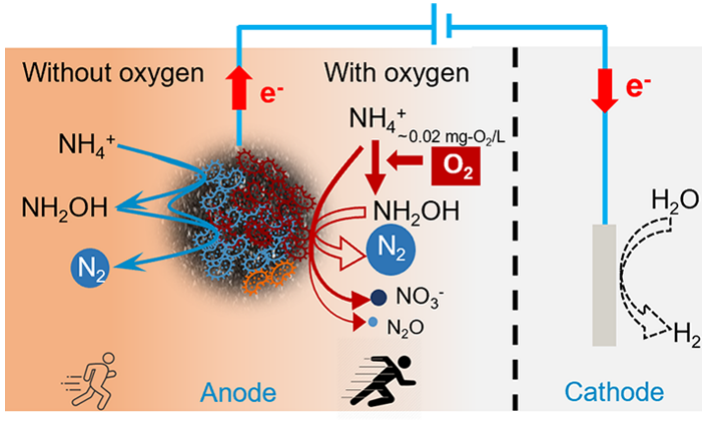

Bioelectrochemical systems (BESs) are considered to be energy-efficient to convert ammonium, which is present in wastewater. The application of BESs as a technology to treat wastewater on an industrial scale is hindered by the slow removal rate and lack of understanding of the underlying ammonium conversion pathways. This study shows ammonium oxidation rates up to 228 ± 0.4 g-N m^–3^ d^–1^ under microoxic conditions (dissolved oxygen at 0.02–0.2 mg-O_2_/L), which is a significant improvement compared to anoxic conditions (120 ± 21 g-N m^–3^ d^–1^). We found that this enhancement was related to the formation of hydroxylamine (NH_2_OH), which is rate limiting in ammonium oxidation by ammonia-oxidizing microorganisms. NH_2_OH was intermediate in both the absence and presence of oxygen. The dominant end-product of ammonium oxidation was dinitrogen gas, with about 75% conversion efficiency in the presence of a microoxic level of dissolved oxygen and 100% conversion efficiency in the absence of oxygen. This work elucidates the dominant pathways under microoxic and anoxic conditions which is a step toward the application of BESs for ammonium removal in wastewater treatment.

## Abstract 2

We achieved high ammonium oxidation rates at bioanodes by introducing low levels of oxygen and revealed the rate limiting step and the pathways of ammonium oxidation at bioanodes.

## Introduction

Nitrogen (N) pollution is a worldwide problem. Increasing N levels in waterbodies cause eutrophication and increase health risks for aquatic ecology and human beings. To avoid these effects, N in wastewater needs to be removed to permissible levels (Total N < 10 mg-N/L) before the water is discharged into the environment.^1,2^ Ammonium (NH_4_^+^) is the major form of N in raw wastewater, accounting for 70%–82% of the Total N concentration.^3^ Nowadays, biological nitrification followed by denitrification is widely applied to removal of NH_4_^+^. During nitrification, ammonia is oxidized by ammonia-oxidizing microorganisms (AOMs) (including ammonia-oxidizing bacteria and archaea) in a cascade of biological reactions to nitrate (NO_3_^–^). In the denitrification step, heterotrophic denitrifiers reduce NO_3_^–^ into dinitrogen gas (N_2_) which subsequently is released into the atmosphere.

For nitrification, wastewater is aerated to supply oxygen, which is, in terms of overall carbon footprint, a challenge. Aeration requires large amounts of energy, i.e. up to 60% of the overall operating expenditures (OPEX).^4^ Furthermore, during aeration, typically 0.1–8.0% of all N in the feed of the wastewater facility is converted in nitrous oxide (N_2_O) by ammonia-oxidizing bacteria, which is a greenhouse gas.^5^ A challenge for denitrification could be the amount of available organic matter. When organic matter is limiting, an external organic carbon source needs to be dosed to the wastewater to secure sufficient denitrification. Due to aforementioned challenges, scientist continuously look for improvements and alternatives for N-removal technologies.^6^ An alternative, energy efficient technology is anaerobic ammonium oxidation (anammox) where NH_4_^+^ is partly oxidized to nitrite (NO_2_^–^), and the remaining NH_4_^+^ is converted to N_2_ using NO_2_^–^ as the electron acceptor.^7^

In recent years, the use of bioelectrochemical systems (BESs) for N removal has received increasing attention. Electrodes may serve as electron donors or acceptors for N metabolism and respiration,^8^ hence avoiding the requirements for addition of organic matter and oxygen as electron donors and acceptors, respectively. Extensive research has proven the concept of NH_4_^+^ oxidization where the anode acts as the electron acceptor.^9−12^ Via this route, the need for an external oxygen supply might be reduced. In the BESs, direct conversion of NH_4_^+^ to N_2_ was obtained; with that, energy input for the further treatment of N intermediates such as NO_2_^–^ or NO_3_^–^ was avoided. Furthermore, full selectivity for N_2_ was reported in anode compartments, i.e. the formation of N_2_O was prevented. Complete removal of NH_4_^+^ to N_2_ was observed at bioanodes with mixed cultures and pure anammox cultures (referred to as electro-anammox), respectively.^12−14^ With these insights, bioelectrochemical systems are a promising selective and energy efficient alternative to conventional N treatment technologies. The reported NH_4_^+^ oxidation rates in these systems were low compared to existing NH_4_^+^ removal technologies, i.e. 10–60 g-N m^–3^ d^–1^ versus 195–351 g-N m^–3^ d^–1^ for nitrification in full-scale wastewater treatment plants (WWTPs).^9−12,15−17^ These low rates require unfavorable large reactor footprints when applied on an industrial scale.

It is essential to understand the complex and variety of pathways of NH_4_^+^ oxidation and find the rate-limiting process factors to improve the NH_4_^+^ oxidation rate at bioanodes with mixed cultures. Vilajeliu-Pons et al. reported nitrifying microorganisms (also known as nitrifiers) as the major contributors to NH_4_^+^ oxidation at the bioanode.^12^ Generally, nitrifiers are considered to be obligate aerobic: they require molecular oxygen for reactions in the N-oxidation pathway and for respiration.^18^ More precisely, in the first step of nitrification, oxygen serves as a substrate, while ammonia is oxidized to NH_2_OH, accompanied by the reduction of oxygen to water. This process is facilitated by the enzyme ammonia monooxygenase.^19^ Complete inhibition of NH_4_^+^ oxidation by AOMs at bioanodes was observed after the addition of Allylthiourea (ATU), which is a commonly used ammonia monooxygenase inhibitor.^12^ However, in the study of Shaw et al., no obvious inhibition of NH_4_^+^ oxidation was observed with pure culture anammox bacteria in the BESs after the addition of ATU.^13^ The results suggest that the oxygen requirements between AOMs and anammox bacteria for NH_4_^+^ oxidation at the electrode are different. This dissimilarity can be attributed to the differential pathways utilized by these microorganisms for NH_4_^+^ oxidation. Nevertheless, it has been shown that NH_2_OH serves as the intermediate for both pathways of NH_4_^+^ oxidation with AOMs and anammox bacteria at bioanodes.^12,13^

Until now, most studies on NH_4_^+^ oxidation with bioanodes avoid the introduction of oxygen, as the presence of oxygen or oxygen compounds reduces the Coulombic efficiency of a BES.^20,21^ Only a few studies investigated the NH_4_^+^ oxidation performance at different dissolved oxygen (DO) concentrations in BESs. It was found that NH_4_^+^ oxidation was faster at higher DO (6.45–3.60 mg-O_2_/L) than at lower DO (2.06 ± 0.33 mg-O_2_/L).^22^ Too low of a DO concentration (0.11–0.29 mg/L) negatively influenced Coulombic efficiency because nitrification was limited by a low DO level.^15^ Even though the DO level is critical for the NH_4_^+^ oxidation rate and Coulombic efficiency, to the best of our knowledge it remains unknown to what extent oxygen influences NH_4_^+^ oxidation pathways and associated rates at the bioanode.

The aim of this study is to enhance the NH_4_^+^ conversion rates at the anode of a BES. Pathways for NH_4_^+^ oxidation were elucidated by manipulating the DO levels in the bioanode with mixed cultures. NH_4_^+^ oxidation rates and end-product formations were derived and compared when a bioanode was operated under microoxic and anoxic conditions. In this study, we use the term “microoxic” to describe a state with a low level of DO, which is achieved by restricting the amount of oxygen supplied.^23^ Conversely, we refer to “anoxic” conditions as those in which no molecule oxygen is available but contain bound oxygen, such as NO_3_^–^ and NO_2_^–^.^24^ In addition, the nitrification inhibitor ATU was dosed to identify the role of AOMs under microoxic and anoxic conditions. Also, the addition of NH_2_OH was carried out to identify the limiting steps during NH_4_^+^ conversion. The results of this study elucidate the importance of oxygen in NH_4_^+^ oxidation at bioanodes, and we discuss the different NH_4_^+^ oxidation pathways.

## Materials and Methods

### 2.1 Reactor Design

**Scheme 1:**
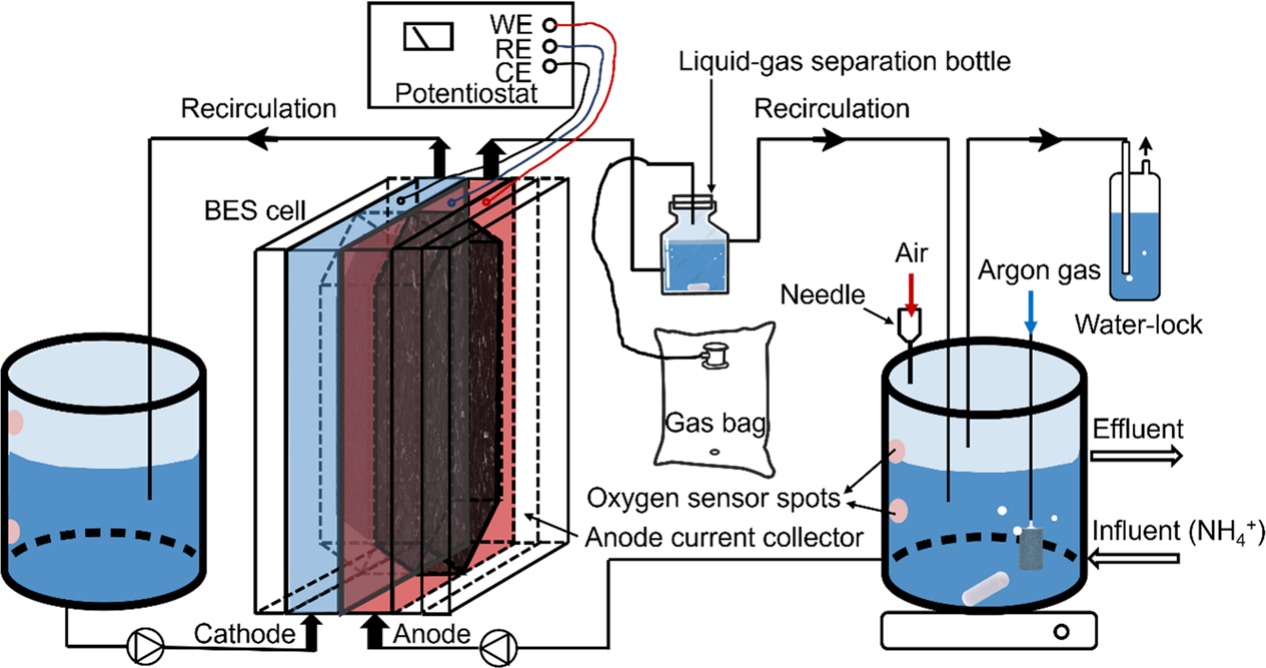
Schematic Diagram of the BES Reactor

Two duplicate dual-chamber BES reactors (Reactor1 and Reactor2, R1 and R2) were constructed identically and operated under the same conditions (Scheme 1). Each BES was a two-chamber reactor with granule activated carbon (GAC) (1–3 mm diameter, Carbot Norit Nederland B.V, Zaandam, The Netherlands) packed anode (R1 with 9.8 g of GAC and R2 with 8.8 g of GAC), where the biofilms attached to and oxidation reactions happen. Graphite felt (21.7 cm^2^) was used for the cathode. Every cell had 30 cm^3^ flow channels in the anodic and cathodic chambers. These two chambers were separated by a bipolar membrane (fumasep FBM-PK, fumatech, Germany) with a projected surface area of 22 cm^2^ with the anion exchange side facing the anode and the cation exchange side facing the cathode. A plain graphite plate with the same working area as the membrane was used as a current collector for the anode. Its purpose was to gather the electrons generated during the oxidation process and transport them to an external circuit. For the cathode, the graphite felt was attached on a titanium wire (grade 2), which served as the current collector. An Ag/AgCl (3 M KCl) reference electrode (+0.205 V vs SHE, QM711X-GEL, ProSense B.V, The Netherlands) was connected to the anolyte solution. All anode potentials are therefore expressed versus Ag/AgCl.

Each anodic chamber was connected to a liquid–gas separation bottle (50 mL) with a gas bag of 500 mL. The NH_4_^+^ solution was pumped into a recirculation bottle (500 mL), which is mixed with the anolyte. The anolyte was recirculated using a two-channel pump (323 process pump, Watson Marlow, UK) shared with both reactors. The overflow liquid of the recirculation bottle was the effluent of the overall reaction system. By piercing the rubber cap of the recirculation bottle with a needle (23G × 1″-NR.16, 0.6 mm × 25.0 mm, BD Microlance 3, USA), the headspace of recirculation bottles of anode was connected to the atmosphere. In that way, oxygen diffused into the anolyte. A porous sparger connected to argon gas was also placed in the recirculation bottle. The recirculation bottle was continuously flushed with argon gas when anoxic conditions are required. The recirculation bottles of the anode were placed on a magnetic stirrer (length 30 mm, diameter 10 mm) and continuously stirred at 120 rpm to provide an even distribution of NH_4_^+^ and oxygen and prevent biomass sediment. The flow rate between the recirculation bottle and anodic chamber was 10 mL/min. The catholyte was also recirculated between a bottle (500 mL) and cathodic chamber with a flow rate of 20 mL/min. At the bioanode, NH_4_^+^ was removed, while abiotic hydrogen was generated at the cathode. The production of hydrogen was shown by the accumulation of H_2_ in the headspace of the cathodic recirculation bottle.

### 2.2 Inoculation and Medium

The anode chambers were inoculated with a mixed sludge from a municipal WWTP of Bennekom, The Netherlands, and a nitrification reactor. The WWTP uses the oxidation ditch technique, and the sludge was collected at the end of the treatment cycle. The nitrification reactor received the same influent as the BES, and the DO was maintained at approximately 2.5 mg-O_2_/L. The biomass concentration of inoculum was 490 ± 10 mg-VSS/L and contained an equal amount of VSS from both sources. A total volume of inocula of 200 mL was added in the recirculation bottles of the anode chamber. The reactors were operated in batch mode until no visible biomass was present in the recirculation bottles and stable NH_4_^+^ oxidation performances were achieved. At day 22, the reactors were switched to continuous mode.

Anode chambers were filled with a NH_4_Cl solution as the reaction substrate, which was prepared by adding 5.0 mL/L of a NH_4_Cl stock solution (10 g of N/L) to a medium solution. The medium solution contained the following components (per liter): NaHCO_3_ 2.00 g, KH_2_PO4 1.36 g, Na_2_HPO_4_ 5.67 g, and 0.50 mL of a trace mineral mix.^25^ No organic carbon was supplied at any time of operation. Cathodic chambers were fed only with medium solution.

**Scheme 2:**
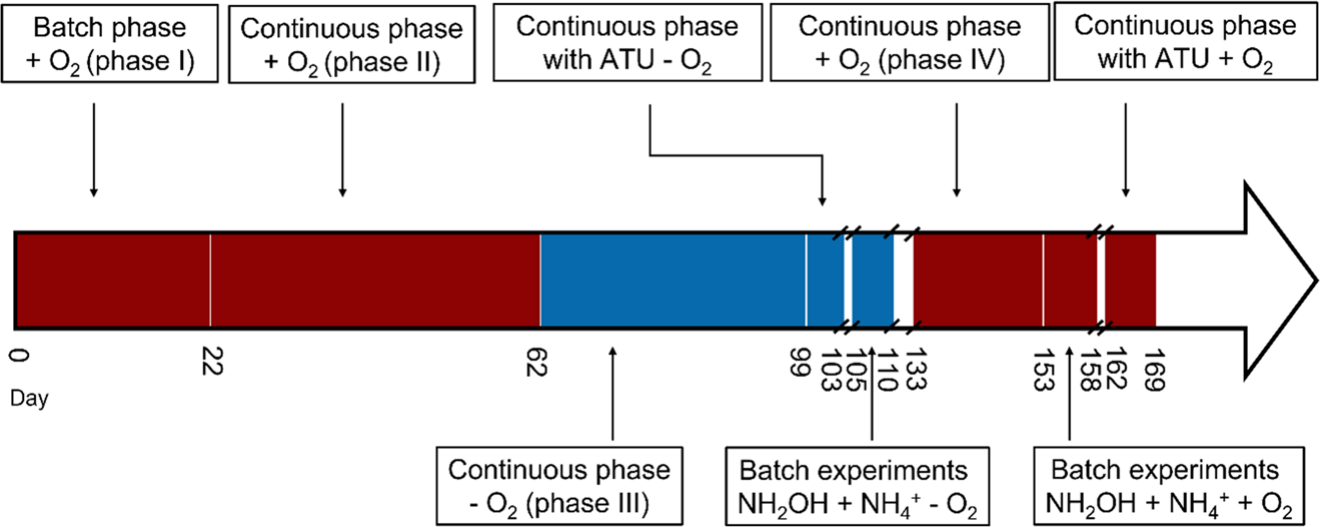
Timeline of the Experiments The red blocks represent the periods with oxygen (+ O_2_), and the blue blocks represent the periods without oxygen (− O_2_). The breaks stand for the periods where reactors stabilized from switching between different conditions or disruption due to sampling. The data collected during these periods were not used in this study.

A series of experiments were conducted in this study, and an overview of the timeline of different experiments can be found in Scheme 2.

### 2.3 Operating Conditions - with and without Oxygen

The batch phase was operated with the presence of oxygen for 22 days, and the subsequent continuous phase was carried out with a microoxic level of oxygen until day 62, followed by an anoxic continuous phase without oxygen until day 132. For the introduction of oxygen, we utilized a needle with a diameter of 0.6 mm and a length of 25.0 mm. This needle was employed to establish a connection between the atmosphere and the headspace of the anodic recirculation bottle. The top of the bottle was sealed with a GL 45 screwcap featuring three GL14 ports. One of these ports was sealed with a rubber cap through which the needle was inserted. Oxygen was provided by passive diffusion to the solution, ensuring a limited oxygen concentration within the system without the need for complex operational and control strategies. To achieve anoxic conditions, the needle was removed at day 62. While a small amount of oxygen was found even when the system was fully closed, the anolyte was constantly flushed with high purity argon gas (99.999%) to maintain anoxic conditions after day 78. The gaseous and liquid oxygen levels were closely monitored. Gaseous oxygen levels were measured and reported as a percentage, while liquid oxygen levels (dissolved oxygen, DO) were measured and reported in units of mg-O_2_/L. The influent of reactors (50 mg-NH_4_–N/L) was sparged with argon gas to eliminate DO. The DO of the analyte remained consistently below the detection limit of 0.015 mg-O_2_/L, and the gaseous oxygen in the anodic recirculation remained below 0.5%.

The catholyte was refreshed every 2 weeks and also flushed with argon gas prior to addition to the recirculation bottle. To ensure the absence of oxygen migration from the cathode to the anode, we measured the DO in the catholyte and the oxygen levels in the headspace of the cathode recirculation. Throughout the continuous phase, the DO remained below the detection limit, and gaseous oxygen levels were consistently below 0.5%. To verify the reversibility of the NH_4_^+^ oxidation performance under different conditions, the experimental setup was adjusted to operate under microoxic conditions since day 133.

To address the uncertainty surrounding the optimal potential for ammonium oxidation, we employed current regulation to establish a favorable anode potential. The reactors were operated at 5 mA from days 0 to 11 using a potentiostat (Ivium n-Stat with IviumSoft v4.990, Eindhoven, The Netherlands). Afterward, the current was reduced to 2 mA (0.09 mA/cm^2^) until the end of the experiments. Anode potentials were measured every minute. The flow rate of influent was 0.15 L d^–1^, corresponding to a nitrogen loading rate of 250 g of N m^–3^ d^–1^. Oxygen levels of the headspace and solution of both recirculation bottles were measured daily. The room temperature was kept constant at 24 ± 1 °C. pH was measured but not controlled. Furthermore, another abiotic experiment was conducted to investigate the DO level of analyte under conditions where oxygen was present, but there was no oxygen consumption from NH_4_^+^ oxidation. Additionally, an abiotic experiment was performed to evaluate the absorption of NH_4_^+^ by GAC. The details of these two experiments are described in the Supporting Information (SI).

### 2.4 Continuous ATU Treatment Experiments

To evaluate the role of AOMs in the NH_4_^+^ oxidation process, the commonly used ammonia monooxygenase inhibitor ATU was used both with and without oxygen. The inhibition experiments were carried out in continuous mode, and ATU was added in the anolyte and in the influent. ATU was added to a final concentration of 50 μM which was determined by a separate experiment (see the SI for details). The anoxic inhibition experiments took place from day 99 to 103, and operation with oxygen took place from day 162 to 169. Liquid samples were taken on a daily basis to analyze the NH_4_^+^ oxidation performance and dissolved N products including NO_2_^–^ and NO_3_^–^.

### 2.5 Batch Operation with NH_2_OH and NH_4_^+^

To assess the role of NH_2_OH in the NH_4_^+^ oxidation pathway, batch tests with the addition of NH_2_OH and NH_4_^+^ were performed at the bioanode. The tests were performed under both micro- and anoxic conditions.

The anode compartment was switched to batch mode, and NH_4_^+^ and NH_2_OH were added in the anode recirculation bottles. The rest of the operations remained the same as continuous phase. The initial NH_2_OH concentration was ∼20 mg-N/L, and the NH_4_Cl concentration was ∼50 mg-N/L. Liquid samples were taken at 0, 10, 30, 60, 120, 240, and 360 min on the first day, and afterward once a day. The dissolved N compounds including NH_2_OH, NH_4_^+^, NO_2_^–^, and NO_3_^–^ were measured, and the potential variations were evaluated. The anoxic batch operation tests were performed from day 105 to 110, and microoxic batch test lasted from day 153 to 158.

### 2.6 Analysis and Calculations

Liquid samples were taken from both anodic and cathodic recirculation bottles and subsequently filtered through a syringe filter (0.2 μm, PES-20/25, CHROMAFIL Xtra, Germany) prior to chemical analysis. Gas samples from the anode were obtained from a gas bag connected to the separation bottle, which was positioned between the BES cell and the recirculation bottle. On the other hand, gas samples from the cathode were collected from the headspace of the cathodic recirculation bottle.

Both gas-phase oxygen concentration and dissolved oxygen were measured with an oxygen Sensor Spot (SP-PSt3-NAU, PreSens, Germany) and oxygen meter (Fibox 4, PreSens, Germany) (measurement range: gaseous oxygen 0–100% O_2_ with limit of detection 0.03%, DO, 0–45 mg-O_2_/L with limit of detection 0.015 mg-O_2_/L). The NH_4_^+^ concentration was determined by a Hach Lange cuvette test (LCK 303 and LCK 305). The NO_2_^–^ and NO_3_^–^ measurements were performed with an ion chromatograph (ICS-2100, Thermo Scientific Dionex, USA) using an analytical column (IonPac AS17-C 2 mm, Thermo Scientific Dionex, USA) and a conductivity detector. Gas samples from the anode were analyzed for the N_2_ and N_2_O concentrations. In contrast, gas samples from the cathode were examined for the H_2_ concentration. The N_2_O concentration was measured with a gas chromatograph (Interscience Trace1300, Thermo Scientific, USA) equipped with an ECD detector and HAYESEP Q column (80–100 1/8″ ss 3m, Agilent, USA), and the N_2_ and H_2_ concentrations were analyzed with a gas chromatograph (Hewlett-Packard 5890, Agilent, USA) equipped with a TCD detector and HP Molsieve 5A column (L × I.D. 30 m × 0.53 mm, film thickness, 25 μm). The NH_2_OH concentration was determined with a colorimetric method as described by Frear et al.^26^ Details about the NO_2_^–^, NO_3_^–^, and NH_2_OH measurements are provided in the SI. The pH was measured using a pH meter (pH M120, Meter Lab, Republic of Korea).

The nitrogen balance was set up by comparing the NH_4_^+^ consumption and other inorganic nitrogen products including NO_2_^–^, NO_3_^–^, N_2_, and N_2_O. N_2_ was only measured in the anoxic phase (III) since the reactor was exposed to atmospheric N_2_ continuously, and the high N_2_ concentrations would not allow for detection of N_2_ as the product of NH_4_^+^ oxidation when operated with oxygen. N_2_ production under microoxic conditions was obtained by closing the balance between removed NH_4_^+^ and the dissolved N and N_2_O products. Both the anolyte and catholyte were sampled for the analysis of the dissolved N compound. A low amount of NH_4_^+^ transferred from the anode to cathode was observed, and no NO_2_^–^ or NO_3_^–^ was detected in the cathode compartments. NH_4_^+^ oxidation efficiency was expressed by the ratio of NH_4_^+^ consumption over the initial NH_4_^+^, and the loss of NH_4_^+^ in the anode compartment due to transfer was subtracted. NH_4_^+^ oxidation rates were expressed relative to the net volume of the anode compartment (30 mL). The electron balance was calculated by comparing the electrons obtained and donated considering the different products (for details, see the SI).

## Results

### 3.1 NH_4_^+^ Oxidation Performance with and without Oxygen

**Figure 1:**
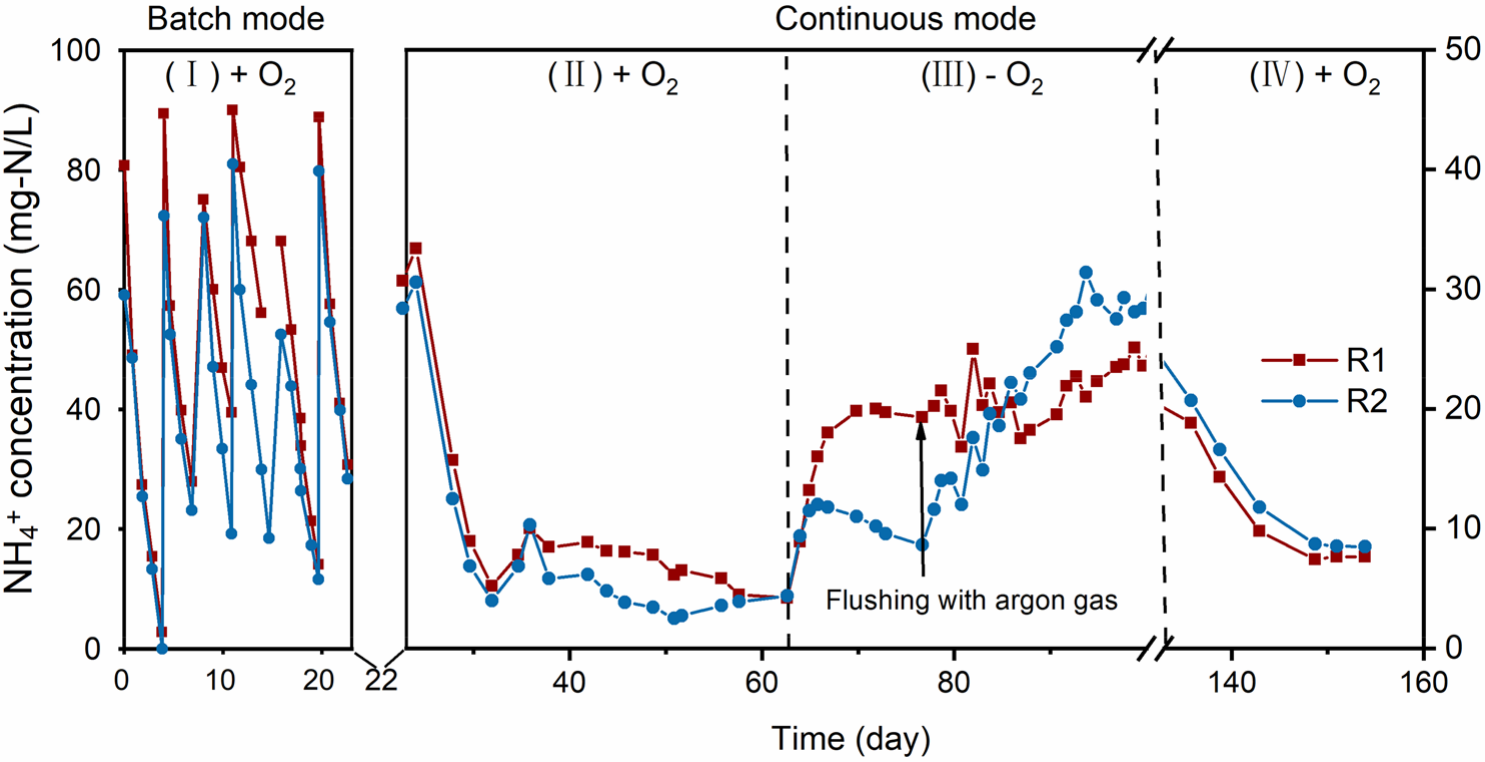
Change of NH_4_^+^ concentrations in the anode compartment of duplicate bioelectrochemical reactors: (I) batch phase (+ O_2_), (II) microoxic continuous phase (+ O_2_), (III) anoxic continuous phase (− O_2_), and (IV) microoxic continuous phase (+ O_2_). An axis break is introduced to remove data points of the experiments in which the aim is not related to this section.

To investigate the influence of oxygen on NH_4_^+^ oxidation at bioanodes, the BESs were operated both with and without oxygen. The two reactors were first operated in batch mode to ensure biofilm attachment on the electrode. The batch tests were carried out with oxygen, including 6 cycles with NH_4_^+^ supplied with a length of 3–4 days per cycle. The NH_4_^+^ oxidation initiated directly after the start of the experiment, shown as decreases in the NH_4_^+^ concentration (Figure 1). The NH_4_^+^ concentration decreased from 80.0 (R1) and 59.0 (R2) mg-N/L to 0 mg-N/L in 5 days. No lag phase was observed, and a stable NH_4_^+^ oxidation performance was obtained during the subsequent cycles.

After switching to continuous operation, the NH_4_^+^ oxidation in the anode rapidly increased and obtained the highest conversion rates within 9 days. At day 37, the NH_4_^+^ concentrations reached a stable low level. Both reactors showed significant NH_4_^+^ oxidation with effluent NH_4_^+^ concentrations as low as 4.3 ± 0.1 mg of N/L (Figure 3). The NH_4_^+^ oxidation efficiency was 91.2 ± 0.2%, and the NH_4_^+^ oxidation rate reached 228 ± 0.4 g of N m^–3^ d^–1^. In an abiotic electrochemical control experiment, no decrease in the NH_4_^+^ concentration over 24 h was observed (Figure S1).

Besides the differences in NH_4_^+^ concentration between the biotic and abiotic experiments, the oxygen levels also differed (Figure S2). In the batch phase, the oxygen level in the bioanode ranged between 0.35 and 2.95 mg-O_2_/L. After switching to the continuous phase, the oxygen continuously decreased and reached a low level of 0.20 mg-O_2_/L at day 62 in the biotic reactors, whereas the oxygen level for the abiotic experiments was considerably higher: 5.76 ± 0.11 mg-O_2_/L.

Starting at day 53, the systems were carefully sealed to avoid exposure to oxygen. A direct increase in NH_4_^+^ concentrations was observed when oxygen was removed, which is an indication of a decrease in NH_4_^+^ oxidation rates (Figure 1, phase III). In R2, decreasing levels of NH_4_^+^ were observed in the anolyte from day 67 onward. Although the DO was under the detection limit (0.015 mg-O_2_/L), the oxygen concentrations of the headspace of R2 increased to 12.0%. To prevent further significant ingress of oxygen, the systems were constantly flushed with argon gas from day 78 onward. Since then, DO remained under the detection limit (0.015 mg-O_2_/L), and oxygen levels in the headspace remained below 0.5% for the rest of phase III. In this study, oxygen production at the anode is unlikely. Anode potentials ranging from 0.52 to 0.55 V were observed, while the calculated potential for O_2_ evolution was 0.50 V versus Ag/AgCl (as determined in the SI). Typically, carbon-based materials require an overpotential exceeding 0.3 V for O_2_ production.^27^ Additionally, a previous abiotic experiment demonstrated the absence of O_2_ evolution at a potential of 0.6 V vs Ag/AgCl.^12^ Under these anoxic conditions, the NH_4_^+^ concentration in the effluent increased to 23.6 and 28.4 mg-N/L for R1 and R2, respectively. On average, the NH_4_^+^ oxidation efficiency decreased to 47.8% ± 8.4%. Reintroducing the oxygen into the anode resulted in immediate decrease of the residual NH_4_^+^ at day 133, and the NH_4_^+^ oxidation activity resumed to the same level as phase II (Figure 1). The DO concentration ranged from 0.02 to 0.05 mg-O_2_/L.

The potentials were recorded and are shown in Figure S3. The potential remained stable under microoxic conditions with a slight increase from 0.52 to 0.55 V vs Ag/AgCl. The potential under anoxic conditions did not change significantly but slightly decreased to 0.52 V vs Ag/AgCl. The pH was stable (around 7.5) during the whole experiment (data not shown).

### 3.2 NH_4_^+^ Oxidation Products with and without Oxygen

To analyze products of the NH_4_^+^ oxidation at a bioanode, dissolved nitrogen, including NO_3_^–^, NO_2_^–^, and N_2_O, was measured. N_2_ production was directly measured only under anoxic conditions. Under microoxic conditions, it was estimated by calculating the difference between the consumed NH_4_^+^ and the generated amounts of NO_2_^–^ and NO_3_^–^, as direct measurement of N_2_ concentration changes was not feasible. However, for future studies, more definitive measurements, such as isotope analysis, will be necessary to accurately assess the nitrogen balance.

**Figure 2:**
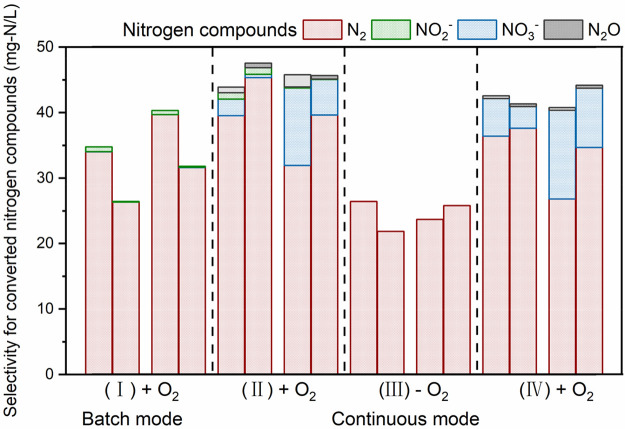
NH_4_^+^ oxidation product distribution with and without oxygen: (I) batch phase (+ O_2_), (II) microoxic continuous phase (+ O_2_), (III) anoxic continuous phase (− O_2_), and (IV) microoxic continuous phase (+ O_2_). The distribution was represented by eight samples which were collected at the beginning and end of each phase. Each set of bars was from both R1 (left bar) and R2 (right bar).

In the presence of oxygen, approximately 75% of oxidized NH_4_^+^ was presumed to be converted into N_2_. Of the remaining products, most were found in the form of NO_3_^–^, while a small part was N_2_O (Figure 2). NO_2_^–^ was found only in a low concentration of 0.41 ± 0.40 mg of N/L in batch mode, and no accumulation was observed throughout the whole experiment. The average concentration of NO_3_^–^ was 0.75 ± 0.43 mg of N/L in batch mode. After changing to continuous operation, accumulation of NO_3_^–^ was observed, reaching a maximum of 11.8 and 5.4 mg-N/L for R1 and R2, respectively. N_2_O measurements were performed on days 52, 55, and 56 (phase II). It was found that 2.1% ± 1.1% of oxidized NH_4_^+^ was converted to N_2_O at the bioanode (Figure 2).

Analysis of N products revealed that no residual NO_2_^–^ and NO_3_^–^ were detected in the effluent under anoxic conditions (phase III). Gas analysis demonstrated that there was no N_2_O production, and complete conversion to N_2_ was obtained (Figure 2). When oxygen was reintroduced to the system, the NO_3_^–^ accumulation and product of N_2_O were observed again. The N_2_O was found to have a similar level as in the previous microoxic period (phase II), while the percentage of NO_3_^–^ increased to 13.4% and 9.1% for R1 and R2 at the end of phase IV. These results show different products for NH_4_^+^ oxidation at the bioanode when a microoxic level of oxygen is present or absent, which indicates the variety of pathways of NH_4_^+^ conversion with and without oxygen present.

### 3.3 The Impact of ATU Addition on NH_4_^+^ Oxidation

To investigate the importance of AOMs for NH_4_^+^ conversion, a set of experiments was performed in which ATU was added to the anolyte with and without oxygen. According to the results of the separate experiment aimed at determining the appropriate ATU concentration, ATU can substantially inhibit the activity of AOMs. No differences of inhibition among the 3 concentrations were observed (50, 100, and 1000 μM) (Figure S4). Therefore, the initial ATU concentration was set at 50 μM. ATU addition experiments were carried out in continuous mode to ensure a constant feed of ATU to the reactor.

**Figure 3:**
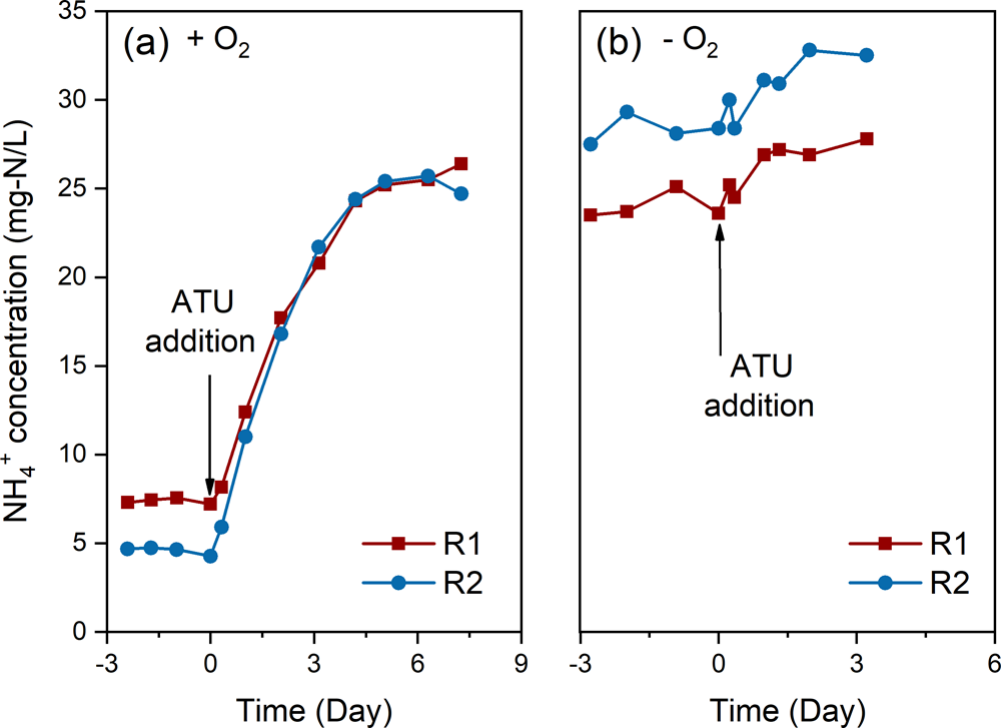
Effect of ATU addition on NH_4_^+^ oxidation in the anode compartment with oxygen (+ O_2_) (a) (days 162 to 169) and without (− O_2_) (b) (days 99 to 103).

Figure 3 shows the NH_4_^+^ concentration in the effluent before and after the addition of ATU in the anolyte. The NH_4_^+^ in the effluent increased from 7.2 to 26.4 mg of N/L and from 4.3 to 24.7 mg of N/L for R1 and R2 after the addition of ATU with oxygen (Figure 3a). DO increased to 1.41 mg-O_2_/L on the first day of ATU addition and decreased to 0.74 mg-O_2_/L at the end of the addition experiments (Figure S2). Under anoxic conditions, on the other hand, no major difference was observed in the NH_4_^+^ concentration of the effluent after adding ATU to the system (Figure 3b). In the anoxic experiments, the average NH_4_^+^ concentration varied from 24.1 ± 0.9 to 26.3 ± 1.6 mg-N/L (R1) and 28.3 ± 0.9 to 30.9 ± 1.6 mg-N/L (R2) due to the addition of ATU. The NH_4_^+^ levels after the addition of ATU were similar to continuous anoxic conditions (phase III).

### 3.4 The Role of Intermediate-NH_2_OH in NH_4_^+^ Oxidation

To identify the role of NH_2_OH, experiments were carried out where NH_2_OH was used as an additional substrate together with NH_4_^+^, both with and without oxygen present. The NH_2_OH addition experiments were performed in batch mode with an initial concentration of ∼20 mg-N/L NH_2_OH and ∼50 mg-N/L NH_4_^+^.

**Figure 4:**
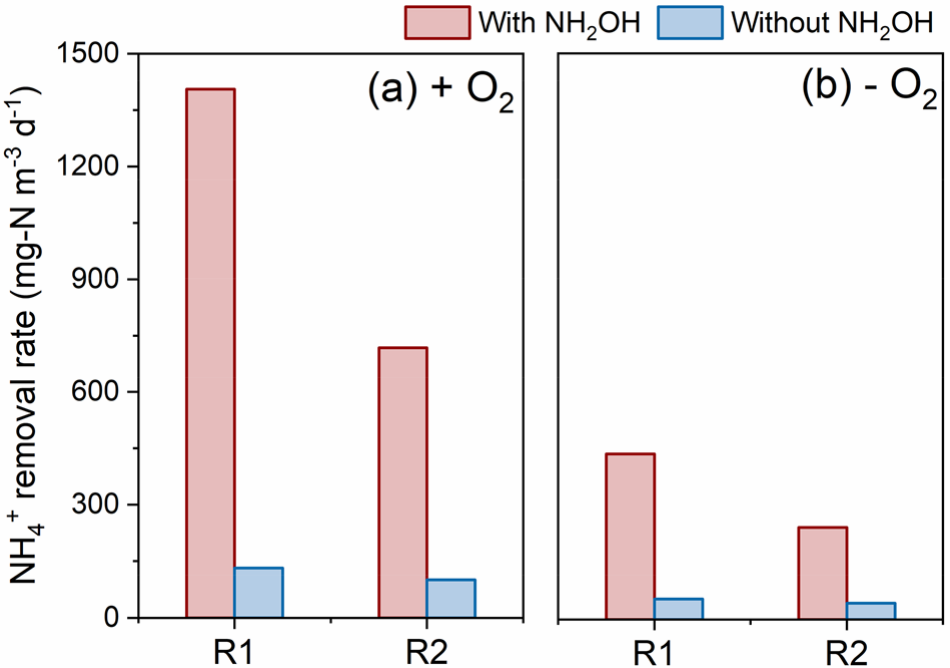
Comparison of NH_4_^+^ oxidation rates in the anode compartment for both with and without NH_2_OH under microoxic conditions (+ O_2_) (a) (days 153 to 158) and under anoxic conditions (− O_2_) (b) (days 105 to 110).

Figure 4 shows the NH_4_^+^ oxidation rate with NH_2_OH and without NH_2_OH for both those with and without oxygen. The NH_4_^+^ oxidation with NH_2_OH was much faster than that without NH_2_OH, which can be explained by the reaction of NH_2_OH with NH_4_^+^ (discussion in section 4.2). The NH_4_^+^ oxidation rate was 1.41 × 10^3^ g-N m^–3^ d^–1^ (R1) and 718 g-N m^–3^ d^–1^ (R2) with NH_2_OH and 132 g-N m^–3^ d^–1^ (R1) and 101 g-N m^–3^ d^–1^ (R2) without NH_2_OH (Figure 4a), observed with a microoxic level of oxygen (DO: 0.02–0.04 mg-O_2_/L). The NH_4_^+^ oxidation rate decreased from 438 to 54 (R1) and from 243 to 43 g-N m^–3^ d^–1^ (R2) when NH_2_OH was depleted without oxygen. The NH_4_^+^ oxidation rates under microoxic conditions remained higher than those under anoxic conditions, which is in accordance with the aforementioned findings. The variation of NH_4_^+^ and NH_2_OH concentrations is shown in Figure S5. NH_2_OH was consumed rapidly once it was added to the anolyte, simultaneously with a significant decrease of NH_4_^+^. The NH_4_^+^ oxidation rate after the depletion of NH_2_OH was restored to a comparable level of the batch phase (phase I). Regarding the products’ formation, upon addition of NH_2_OH during NH_4_^+^ oxidation, we observed the accumulation of NO_2_^–^ to low concentration levels (i.e., ∼1 mg of N/L), which exhibited a rapid disappearance and subsequent accumulation of NO_3_^–^. In contrast, under anoxic conditions, we detected neither NO_2_^–^ nor NO_3_^–^ in the analyte (data not shown).

Figure S3 shows the changes in the potential during the experiments. The anode potential decreased immediately from 0.52 to 0.45 V in 0.19 days after the addition of NH_2_OH. It increased to 0.53 V in 1.91 days under conditions without oxygen. While in the presence of oxygen, the potential decreased from 0.56 to 0.41 V in 0.13 days and recovered to 0.54 V in 1.75 days.

## Discussion

### 4.1 Oxygen Improves the NH_4_^+^ Oxidation Rate at the Bioanode

Complete autotrophic NH_4_^+^ oxidation at the bioanode where an electrode served as an electron acceptor was recently shown.^9,12,14^ It combines low energy consumption and high efficiency of conversion to harmless products (N_2_).^28^ However, the rather slow removal rate and lack of understanding of the underlying NH_4_^+^ conversion pathways at bioanodes hinder the application of this technology.

This study revealed that providing a microoxic level of oxygen in the bioanode can substantially increase the NH_4_^+^ oxidation rate. The NH_4_^+^ oxidation rates were 228 ± 0.4 g-N m^–3^ d^–1^ and 120 ± 21 g-N m^–3^ d^–1^, respectively, under microoxic and anoxic conditions. NH_4_^+^ oxidation efficiency was 91.4 ± 0.2% under microoxic conditions (DO ranged from 0.02 to 0.20 mg-O_2_/L), while it was 47.8% ± 8.4% when the oxygen was absent. Thus, the presence of oxygen boosts the NH_4_^+^ oxidation at bioanode.

The promotion of NH_4_^+^ oxidation by oxygen at the bioanode is striking. It can be attributed to the role of oxygen as the electron acceptor in the initial step of oxidizing NH_4_^+^ to NH_2_OH, which represents the rate limiting step for the nitrification process.^29−31^ Vilajeliu-Pons et al. reported that nitrifying microorganisms performed NH_4_^+^ oxidation at the anode.^12^ AOMs are the most common NH_4_^+^ oxidizers, and it is known that NH_4_^+^ oxidation by AOMs is an oxygen-dependent process.^18^ Stenstrom and Poduska reviewed the influence of oxygen on the AOM activity and found that the NH_4_^+^ concentration decreased fast when the oxygen level was high.^32^ The requirement of oxygen was also confirmed by thermodynamic calculations.^22^ Therefore, NH_4_^+^ oxidation is restricted by AOMs when oxygen availability is limited.

Further relation between the oxygen dependence of NH_4_^+^ oxidation and nitrifying activity can be found in ATU addition experiments, in which ATU inhibits nitrifying activity. The addition of ATU resulted in a decrease in NH_4_^+^ oxidation with oxygen from 88.5% ± 4.0% to 50.0% ± 2.5%, which is comparable to the oxidation level observed during anoxic conditions (47.8% ± 8.4%). However, ATU addition affected the NH_4_^+^ anoxic oxidation significantly less (Figure 3). The results from the ATU tests were consistent with our hypothesis that the decreased NH_4_^+^ oxidation rates with oxygen compared to those without oxygen at the bioanode are due to the inhibition of AOM activity, to be specific, the inhibition of ammonia monooxygenase (NH_4_^+^ to NH_2_OH).^33^ We thus hypothesize that the higher NH_4_^+^ oxidation rate in the presence of oxygen is caused by the AOM activity.

The Coulombic efficiency (CE) was notably higher compared with the other studies. We acquired a CE of 92.3 ± 4.2% under microoxic conditions and 221 ± 23.1% under anoxic conditions. This high CE may be attributed to hydrogen crossover from the cathode. The proximity of the anode and cathode allows for the transfer of hydrogen between the compartments. This phenomenon might be reflected in the low hydrogen yield obtained at the cathode, ranging from 4.8 to 20.3%. It suggests that the unaccounted hydrogen may have undergone transfer to the anode compartment. In a previous study, an artificially high Coulombic efficiency (ranging from 190% to 310%) was reported, attributed to the transfer of hydrogen from the cathode to the anode, serving as an electron donor for anode-respiring bacteria.^34^ When the CE is calculated in this study, the presence of hydrogen crossover in the system introduces uncertainties and challenges. Therefore, the CE results in this study should be interpreted with caution and are not considered as a quantitative guideline.

### 4.2 NH_4_^+^ Oxidation Pathway at a Bioanode

The pathway of NH_4_^+^ oxidation at a bioanode has not been studied extensively. This study provides novel insights into the underlying processes behind NH_4_^+^ oxidation to N_2_. The different NH_4_^+^ oxidation performances and product distribution under microoxic and anoxic conditions suggest there are different pathways for NH_4_^+^ oxidation. The fast switch of these trends after changing dissolved oxygen regimes shows the reversibility of the pathways.

**Scheme 3:**
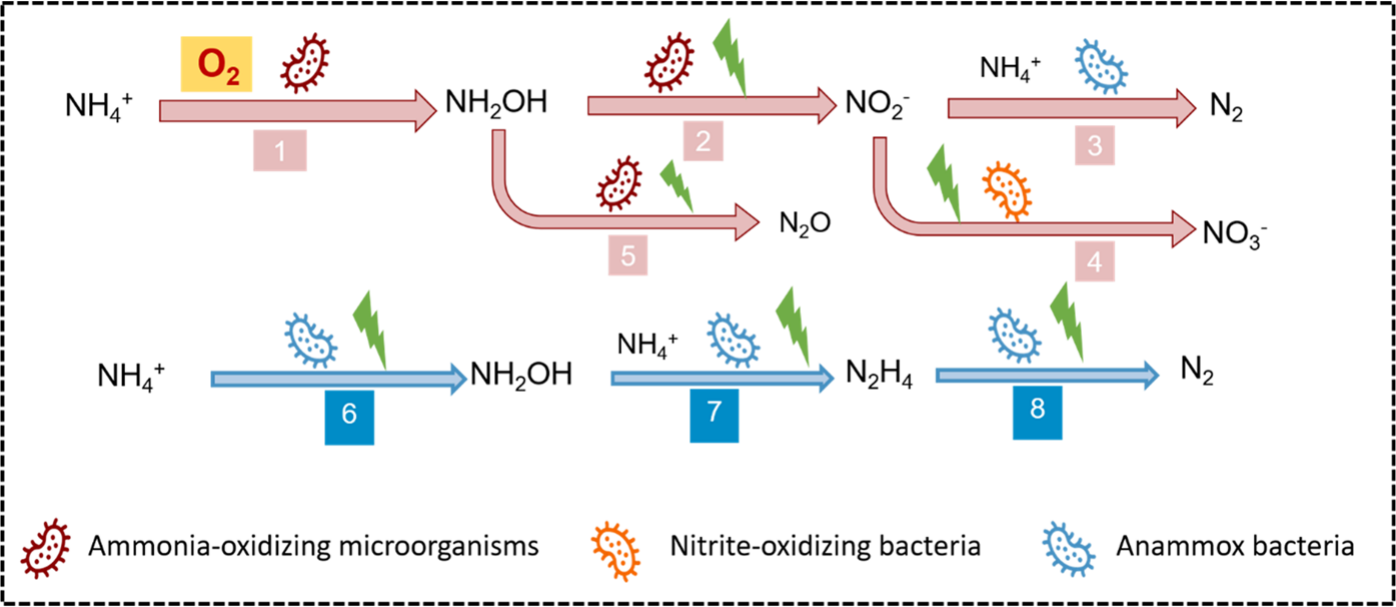
Proposed Pathways of NH_4_^+^ Oxidation at a Bioanode Pink and blue arrows indicate the reactions take place under microoxic and anoxic conditions. The green flash icons indicate the potential processes in which the electrode has been involved. Under microoxic conditions, they are (1) NH_4_^+^ oxidation to NH_2_OH by AOMs with oxygen as the electron acceptor; (2) bioelectrochemical NH_2_OH oxidation to NO_2_^–^; (3) NO_2_^–^ was removed, and the product was N_2_, presumably by the traditional anammox; (4) NO_2_^–^ was oxidized to NO_3_^–^ via nitration by nitrite-oxidizing bacteria; (5) (bio) electrochemical NH_2_OH oxidation to N_2_O; under anoxic conditions, electro-anammox is likely the main activity; (6) NH_4_^+^ oxidation to NH_2_OH by anammox bacteria with the electrode as the electron acceptor; (7) NH_2_OH was condensed with NH_4_^+^ to produce N_2_H_4_; (8) oxidation of N_2_H_4_ to N_2_.

Based on the findings in this study, two main pathways are proposed for NH_4_^+^ oxidation to N_2_ at a bioanode with and without oxygen: 1) nitritation combined with anammox (pink arrow pathway in Scheme 3) and 2) electro-anammox (blue arrow pathway in Scheme 3). NH_2_OH is an important intermediate in both pathways. In the microoxic state, it is an integrated process with AOMs in collaboration with anammox bacteria. AOMs can oxidize NH_4_^+^ to NH_2_OH with oxygen as the electron acceptor. Subsequently, NH_2_OH can be oxidized to NO_2_^–^ and NO_3_^–^, and these may serve as electron acceptors for oxidation of NH_4_^+^ to N_2_ by anammox bacteria. During the NH_2_OH oxidation process, there is a competition between the O_2_ and electrode as potential electron acceptors. However, due to the limited availability of O_2_ and the thermodynamic feasibility of NH_2_OH oxidation (+0.3 V vs Ag/AgCl),^35^ the electrode is more likely to be involved in the oxidation of NH_2_OH. Furthermore, it is suggested that the oxidation of NH_2_OH predominantly occurs through bioelectrochemical oxidation rather than solely electrochemical oxidation.^14^ This is also supported by the observation in the NH_2_OH addition experiments that the provision of NH_2_OH leads to a decrease in the anode potential, indicating its involvement in the bioelectrochemical processes.

While a microoxic level of oxygen was present, small amounts of NO_2_^–^, NO_3_^–^, and N_2_O were detected. Part of the NH_4_^+^ oxidation pathway was inhibited by adding ATU. ATU is a common inhibitor for the conversion of NH_4_^+^ to NH_2_OH by AOMs,^33^ which means that part of the NH_4_^+^ oxidation to NH_2_OH was conducted with oxygen by AOMs. The addition of NH_2_OH also significantly increased the NH_4_^+^ oxidation rate under microoxic conditions. This reveals that NH_2_OH is the intermediate for NH_4_^+^ oxidation via reduction of oxygen. It is postulated that the primary formation of N_2_O occurs during the process of NH_2_OH oxidation. Nitrifier denitrification, involving NO_2_^–^ reduction by nitrifiers, is less likely under the observed anode potentials (0.52–0.55 V vs Ag/AgCl), which are considerably higher than the theoretical reduction potential of NO_2_^–^ to NO (0.17 V vs Ag/AgCl at pH = 7).^36^ The fact that NH_2_OH addition enhances NH_4_^+^ oxidation is related to the conversion pathways. In the presence of oxygen, NH_4_^+^ reacts together with NO_2_^–^ to N_2_, and thus, the presence of NH_2_OH leads to an increased NO_2_^–^ formation oxidation rate at the bioanode, which subsequently promotes the NH_4_^+^ conversion rate (Scheme 3, pathway 3). It is likely that a mixture of anammox related reactions uses NO_3_^–^ and NO_2_^–^, e.g., anammox bacteria could utilize NO_2_^–^ from AOMs to oxidize NH_4_^+^.

The pathway for NH_4_^+^ oxidation in the presence of the microoxic level of oxygen is different from the pathway for NH_4_^+^ oxidation in the absence of oxygen. In the absence of oxygen, no intermediate metabolites such as NO_2_^–^, NO_3_^–^, or N_2_O were detected, and ATU did not appear to inhibit the NH_4_^+^ oxidation. The presence of NH_2_OH significantly stimulates the NH_4_^+^ oxidation rate. These results of the anoxic phases support electroactivity of anammox bacteria, which was referred to as electro-anammox in earlier studies.^13,37,38^ In electro-annamox, NH_2_OH is also the intermediate. The reaction of NH_2_OH at the bioanode differs from further oxidation. Instead, the following reaction of NH_4_^+^ and NH_2_OH leads to the production of hydrazine (N_2_H_4_), and subsequent oxidation of N_2_H_4_ leads to the formation of N_2_ (Scheme 3, pathways 6–8).^13^ However, this process is slower compared to the cooperative mechanism involving AOMs and anammox, which explains why NH_4_^+^ oxidation rates remained lower than those under microoxic conditions.

### 4.3 Perspective of NH_4_^+^ Oxidization Using BES

This study provides a novel perspective for further development of NH_4_^+^ oxidation at the bioanode by operating under microoxic conditions. Compared with a previous study with BES technology, the removal rate is up to 7 times higher, and in addition, NH_4_^+^ levels can be decreased to ∼3 mg-N/L. The performances of different technologies are summarized in Table 1. Strikingly, the NH_4_^+^ oxidation rate is comparable to the rate in full-scale biological nutrient removal WWTPs.^17^ In our study, the oxygen requirement is much lower than in traditional nitrification, in which 2 mol of oxygen per mole of NH_4_^+^ are typically required.^39^ However, in our system, the oxidation of NH_2_OH could occur with the electrode as electron acceptor instead of oxygen. As a result, only 1 mol of oxygen was needed for this conversion, leading to a significant decrease in the energy demand. The NH_4_^+^ oxidation rates obtained in this study are comparatively lower than those reported for the anammox (Table 1). However, oxidation at a bioanode offers advantages in terms of effective control of the system during start-up and stability of operation.^40^

**Table 1:** Comparison of the NH_4_^+^ Oxidation Performance between Conventional Treatment Technologies, Existing Bioelectrochemical Technologies, and Micro-Oxygen Technology

Technology	Conditions	NH_4_^+^ oxidation products	NH_4_^+^ oxidation efficiency (%)	NH_4_^+^ oxidation rate (g-N/m^3^*d)	Ref
Nitrification	Aeration	NO_3_^–^, NO_2_, N_2_O	-	195–351	(17)
Complete nitrification/denitrification	Aeration, additional carbon resource	N_2_, NO_3_^–^, N_2_O	88	112	(41)
Anammox	Appropriate NO_2_^–^ to NH_4_^+^ ratio	N_2,_ N_2_O, NO_3_^–^	95	250–2.73 × 10^5^	(42, 43)
Cathode-denitrification	Additional process for nitrification	N_2_	-	126	(44)
Bioanode NH_4_^+^ oxidation	No aeration, potentiostatical controlled	N_2_	32 ± 5	35 ± 10	(12)
Electro-anammox potentiostatical controlled	No aeration	N_2_	88	∼ 5	(13)
Micro-Oxygen bioanode NH_4_^+^ oxidation	No active aeration	Mainly N_2_, small amount of NO_3_^–^ and N_2_O	93	162–250	This study
	Galvanostaticall/potentiostatically controlled				

Small amounts of N_2_O were detected under microoxic conditions, accounting for ∼2% of the NH_4_^+^ loading. The level is in the same range as in existing technologies such as nitrification-denitrification and nitritation-anammox.^45^ The production of N_2_O can be reduced through improved plant design and operation, for instance, regulating the oxygen supply or adjusting the anode potential.^46,47^ Alternatively, it has been reported that N_2_O can be reduced at the biocathode to obtain a cleaner technology by combining the anode and cathode.^48^

In our study, high electro-anammox activity was observed with a mixed culture in this study, while no results on microbial community composition were obtained here. In previous bioanode studies, anammox bacteria were only found at low abundance.^12,14^ The NH_4_^+^ oxidation rate is higher than the pure culture electro-anammox activity (Table 1), indicating that having synergistic relations between microbial groups in mixed culture is beneficial for NH_4_^+^ removal. It is possible to hypothesize that such connections exist between AOMs and anammox bacteria. The introduction of oxygen favored the activity of AOMs and provided electron acceptors such as NO_2_^–^ for anammox bacteria to oxidize NH_4_^+^.^49^ During the start-up phase, rapid NH_4_^+^ reduction was observed, accompanied by immediate initiation of NH_4_^+^ oxidation. In contrast, a study without oxygen reported a delayed onset of ammonium oxidation on day 17.^12^ This finding suggests that the presence of oxygen significantly accelerated the start-up period for NH_4_^+^ oxidation at the bioanode. The cooperation of AOMs and anammox at a working electrode makes this process easier and faster to start-up.

The results clearly show that NH_4_^+^ oxidation using a bioanode with oxygen achieved high activities. Although our objective did not involve targeting a specific oxygen level, further investigation is warranted to study the impact of oxygen on bioanodic ammonium oxidation. Elucidating the underlying reactions involving oxygen and the electrode would provide valuable insights and contribute to advancing the understanding of this process. NO_3_^–^ accumulation and small amounts of N_2_O produced were observed, so future studies should also focus on the prevention or elimination of these undesired products.

## Supporting Information Available

Figures of supplemental experiment results, including abiotic NH_4_^+^ absorption (Figure S1), dissolved oxygen variation (Figure S2), evolutions of anode potential (Figure S3), batch tests with different ATU concentrations (Figure S4), batch tests with NH_2_OH and NH_4_^+^ (Figure S5), and additional experimental details and methods (PDF)

## ABBREVIATIONS

AOB: ammonia-oxidizing bacteria
anammox: anaerobic ammonium oxidation
BESs: bioelectrochemical systems
WWTPs: wastewater treatment plants
AOMs: ammonia-oxidizing microorganisms
DO: dissolved oxygen
GAC: granular activated carbon
CE: Coulombic efficiency

## References

[ref1] Rahimi S.; Modin O.; Mijakovic I. Technologies for biological removal and recovery of nitrogen from wastewater. Biotechnol. Adv. 2020, 43, 107570 10.1016/j.biotechadv.2020.107570. 32531318

[ref2] Directorate-General for Environment, In Urban Waste Water Directive 98/15/EEC; European Commission: 1998. https://eur-lex.europa.eu/eli/dir/1998/15/oj (accessed 2023-07-17).

[ref3] Li Y.-h.; Li H.-b.; Xu X.-y.; Xiao S.-y.; Wang S.-q.; Xu S.-c. Fate of nitrogen in subsurface infiltration system for treating secondary effluent. Water Sci. Eng. 2017, 10 (3), 217–224. 10.1016/j.wse.2017.10.002.

[ref4] Nancharaiah Y. V.; Venkata Mohan S.; Lens P. N. L. Recent advances in nutrient removal and recovery in biological and bioelectrochemical systems. Bioresour. Technol. 2016, 215, 173–185. 10.1016/j.biortech.2016.03.129. 27053446

[ref5] Prosser J. I.; Hink L.; Gubry-Rangin C.; Nicol G. W. Nitrous oxide production by ammonia oxidizers: physiological diversity, niche differentiation and potential mitigation strategies. Global Change Biol. 2020, 26 (1), 103–118. 10.1111/gcb.14877. 31638306

[ref6] Chan-Pacheco C. R.; Valenzuela E. I.; Cervantes F. J.; Quijano G. Novel biotechnologies for nitrogen removal and their coupling with gas emissions abatement in wastewater treatment facilities. Sci. Total Environ. 2021, 797, 149228 10.1016/j.scitotenv.2021.149228. 34346385

[ref7] Kuenen J. G. Anammox bacteria: from discovery to application. Nat. Rev. Microbiol. 2008, 6 (4), 320–326. 10.1038/nrmicro1857. 18340342

[ref8] Gregory K. B.; Bond D. R.; Lovley D. R. Graphite electrodes as electron donors for anaerobic respiration. Environ. Microbiol. 2004, 6 (6), 596–604. 10.1111/j.1462-2920.2004.00593.x. 15142248

[ref9] Qu B.; Fan B.; Zhu S.; Zheng Y. Anaerobic ammonium oxidation with an anode as the electron acceptor. Environ. Microbiol. Rep. 2014, 6 (1), 100–5. 10.1111/1758-2229.12113. 24596267

[ref10] Zhan G.; Li D.; Tao Y.; Zhu X.; Zhang L.; Wang Y.; He X. Ammonia as carbon-free substrate for hydrogen production in bioelectrochemical systems. Int. J. Hydrogen Energy 2014, 39 (23), 11854–11859. 10.1016/j.ijhydene.2014.05.176.

[ref11] Zhan G.; Zhang L.; Tao Y.; Wang Y.; Zhu X.; Li D. Anodic ammonia oxidation to nitrogen gas catalyzed by mixed biofilms in bioelectrochemical systems. Electrochim. Acta 2014, 135, 345–350. 10.1016/j.electacta.2014.05.037.

[ref12] Vilajeliu-Pons A.; Koch C.; Balaguer M. D.; Colprim J.; Harnisch F.; Puig S. Microbial electricity driven anoxic ammonium removal. Water Res. 2018, 130, 168–175. 10.1016/j.watres.2017.11.059. 29220717

[ref13] Shaw D. R.; Ali M.; Katuri K. P.; Gralnick J. A.; Reimann J.; Mesman R.; van Niftrik L.; Jetten M. S. M.; Saikaly P. E. Extracellular electron transfer-dependent anaerobic oxidation of ammonium by anammox bacteria. Nat. Commun. 2020, 11 (1), 2058 10.1038/s41467-020-16016-y. 32345973 PMC7188810

[ref14] Osset-Álvarez M.; Pous N.; Chiluiza-Ramos P.; Bañeras L.; Balaguer M. D.; Puig S. Unveiling microbial electricity driven anoxic ammonium removal. Bioresour. Technol. Rep. 2022, 17, 100975 10.1016/j.biteb.2022.100975.

[ref15] Zhan G.; Zhang L.; Li D.; Su W.; Tao Y.; Qian J. Autotrophic nitrogen removal from ammonium at low applied voltage in a single-compartment microbial electrolysis cell. Bioresour. Technol. 2012, 116, 271–7. 10.1016/j.biortech.2012.02.131. 22572551

[ref16] He Z.; Kan J.; Wang Y.; Huang Y.; Mansfeld F.; Nealson K. H. Electricity production coupled to ammonium in a microbial fuel cell. Environ. Sci. Technol. 2009, 43 (9), 3391–3397. 10.1021/es803492c. 19534163

[ref17] Yao Q.; Peng D.-C. Nitrite oxidizing bacteria (NOB) dominating in nitrifying community in full-scale biological nutrient removal wastewater treatment plants. AMB Express 2017, 7 (1), 25 10.1186/s13568-017-0328-y. 28116698 PMC5256632

[ref18] Gruber N. The Marine Nitrogen Cycle: Overview and Challenges. In Nitrogen in the Marine Environment, 2nd ed.;Capone D. G., Bronk D. A., Mulholland M. R., Carpenter E. J., Eds.; Academic Press: 2008; pp 1–50.

[ref19] Arp D. J.; Sayavedra-Soto L. A.; Hommes N. G. Molecular biology and biochemistry of ammonia oxidation by Nitrosomonas europaea. Arch. Microbiol. 2002, 178, 250–255. 10.1007/s00203-002-0452-0. 12209257

[ref20] Logan B. E.; Call D.; Cheng S.; Hamelers H. V.; Sleutels T. H.; Jeremiasse A. W.; Rozendal R. A. Microbial electrolysis cells for high yield hydrogen gas production from organic matter. Environ. Sci. Technol. 2008, 42 (23), 8630–8640. 10.1021/es801553z. 19192774

[ref21] Kargi F.; Eker S. Electricity generation with simultaneous wastewater treatment by a microbial fuel cell (MFC) with Cu and Cu-Au electrodes. J. Chem. Technol. Biotechnol. 2007, 82 (7), 658–662. 10.1002/jctb.1723.

[ref22] Xie Z.; Chen H.; Zheng P.; Zhang J.; Cai J.; Abbas G. Influence and mechanism of dissolved oxygen on the performance of Ammonia-Oxidation Microbial Fuel Cell. Int. J. Hydrogen Energy 2013, 38 (25), 10607–10615. 10.1016/j.ijhydene.2013.06.056.

[ref23] Morris R. L.; Schmidt T. M. Shallow breathing: bacterial life at low O2. Nat. Rev. Microbiol. 2013, 11 (3), 205–212. 10.1038/nrmicro2970. 23411864 PMC3969821

[ref24] Sperling M. v. Basic principles of wastewater treatment; IWA Publishing: London, UK, 2007; p 19.

[ref25] Balch W. E.; Fox G. E.; Magrum L. J.; Woese C. R.; Wolfe R. S. Methanogens: reevaluation of a unique biological group. Microbiol. Rev. 1979, 43 (2), 260–96. 10.1128/mr.43.2.260-296.1979. 390357 PMC281474

[ref26] Frear D. S.; Burrell R. C. Spectrophotometric Method for Determining Hydroxylamine Reductase Activity in Higher Plants. Anal. Chem. 1955, 27 (10), 1664–1665. 10.1021/ac60106a054.

[ref27] Noor T.; Yaqoob L.; Iqbal N. Recent Advances in Electrocatalysis of Oxygen Evolution Reaction using Noble-Metal, Transition-Metal, and Carbon-Based Materials. ChemElectroChem. 2021, 8 (3), 447–483. 10.1002/celc.202001441.

[ref28] Su D.; Chen Y. Advanced bioelectrochemical system for nitrogen removal in wastewater. Chemosphere 2022, 292, 133206 10.1016/j.chemosphere.2021.133206. 34922956

[ref29] Caranto J. D.; Lancaster K. M. Nitric oxide is an obligate bacterial nitrification intermediate produced by hydroxylamine oxidoreductase. Proc. Natl. Acad. Sci. U. S. A. 2017, 114, 8217 10.1073/pnas.1704504114. 28716929 PMC5547625

[ref30] Zhu X.; Burger M.; Doane T. A.; Horwath W. R. Ammonia oxidation pathways and nitrifier denitrification are significant sources of N2O and NO under low oxygen availability. Proc. Natl. Acad. Sci. U. S. A. 2013, 110 (16), 6328–33. 10.1073/pnas.1219993110. 23576736 PMC3631630

[ref31] Newell S. E.; Babbin A. R.; Jayakumar A.; Ward B. B. Ammonia oxidation rates and nitrification in the Arabian Sea. Global Biogeochem. Cycles 2011, 25 (4), GB4016 10.1029/2010GB003940.

[ref32] Stenstrom M. K.; Poduska R. A. The effect of dissolved oxygen concentration on nitrification. Water Res. 1980, 14 (6), 643–649. 10.1016/0043-1354(80)90122-0.

[ref33] Bédard C.; Knowles R. Physiology, biochemistry, and specific inhibitors of CH4, NH4+, and CO oxidation by methanotrophs and nitrifiers. Microbiol. Rev. 1989, 53 (1), 68–84. 10.1128/mr.53.1.68-84.1989. 2496288 PMC372717

[ref34] Lee H.-S.; Rittmann B. E. Significance of Biological Hydrogen Oxidation in a Continuous Single-Chamber Microbial Electrolysis Cell. Environ. Sci. Technol. 2010, 44 (3), 948–954. 10.1021/es9025358. 20030379

[ref35] Goolsby A. D.; Sawyer D. T. The electrochemical oxidation of hydroxylamine at platinum and gold electrodes in dimethylsulfoxide. J. Electroanal. Chem. Interfacial Electrochem. 1968, 19 (4), 405–411. 10.1016/S0022-0728(68)80103-2.

[ref36] Hosseininasab V.; DiMucci I. M.; Ghosh P.; Bertke J. A.; Chandrasekharan S.; Titus C. J.; Nordlund D.; Freed J. H.; Lancaster K. M.; Warren T. H. Lewis acid-assisted reduction of nitrite to nitric and nitrous oxides via the elusive nitrite radical dianion. Nat. Chem. 2022, 14 (11), 1265–1269. 10.1038/s41557-022-01025-9. 36064970 PMC9633411

[ref37] Joicy A.; Song Y. C.; Yu H.; Chae K. J. Nitrite and nitrate as electron acceptors for bioelectrochemical ammonium oxidation under electrostatic field. J. Environ. Manage. 2019, 250, 109517 10.1016/j.jenvman.2019.109517. 31545180

[ref38] Yin X.; Qiao S.; Zhou J.; Quan X. Using three-bio-electrode reactor to enhance the activity of anammox biomass. Bioresour. Technol. 2015, 196, 376–82. 10.1016/j.biortech.2015.07.096. 26255601

[ref39] Soler-Jofra A.; Perez J.; van Loosdrecht M. C. M. Hydroxylamine and the nitrogen cycle: A review. Water Res. 2021, 190, 116723 10.1016/j.watres.2020.116723. 33352529

[ref40] Nsenga Kumwimba M.; Lotti T.; Senel E.; Li X.; Suanon F. Anammox-based processes: How far have we come and what work remains? A review by bibliometric analysis. Chemosphere 2020, 238, 124627 10.1016/j.chemosphere.2019.124627. 31548173

[ref41] Lang X.; Li Q.; Ji M.; Yan G.; Guo S. Isolation and niche characteristics in simultaneous nitrification and denitrification application of an aerobic denitrifier, Acinetobacter sp. YS2. Bioresour. Technol. 2020, 302, 122799 10.1016/j.biortech.2020.122799. 31981809

[ref42] Cho S.; Kambey C.; Nguyen V. Performance of Anammox Processes for Wastewater Treatment: A Critical Review on Effects of Operational Conditions and Environmental Stresses. Water 2020, 12 (1), 20 10.3390/w12010020.

[ref43] Isanta E.; Bezerra T.; Fernández I.; Suárez-Ojeda M. E.; Pérez J.; Carrera J. Microbial community shifts on an anammox reactor after a temperature shock using 454-pyrosequencing analysis. Bioresour. Technol. 2015, 181, 207–213. 10.1016/j.biortech.2015.01.064. 25656864

[ref44] Al-Mamun A.; Jafary T.; Baawain M. S.; Rahman S.; Choudhury M. R.; Tabatabaei M.; Lam S. S. Energy recovery and carbon/nitrogen removal from sewage and contaminated groundwater in a coupled hydrolytic-acidogenic sequencing batch reactor and denitrifying biocathode microbial fuel cell. Environ. Res. 2020, 183, 109273 10.1016/j.envres.2020.109273. 32105886

[ref45] Kampschreur M. J.; Temmink H.; Kleerebezem R.; Jetten M. S.; van Loosdrecht M. C. Nitrous oxide emission during wastewater treatment. Water Res. 2009, 43 (17), 4093–103. 10.1016/j.watres.2009.03.001. 19666183

[ref46] Peng L.; Ni B. J.; Erler D.; Ye L.; Yuan Z. The effect of dissolved oxygen on N2O production by ammonia-oxidizing bacteria in an enriched nitrifying sludge. Water Res. 2014, 66, 12–21. 10.1016/j.watres.2014.08.009. 25179869

[ref47] Jebaraj A. J. J.; Kumsa D.; Scherson D. A. Oxidation of Hydroxylamine on Gold Electrodes in Aqueous Electrolytes: Rotating Ring-Disk and In Situ Infrared Reflection Absorption Spectroscopy Studies. J. Phys. Chem. C 2012, 116 (12), 6932–6942. 10.1021/jp2104566.

[ref48] Desloover J.; Puig S.; Virdis B.; Clauwaert P.; Boeckx P.; Verstraete W.; Boon N. Biocathodic nitrous oxide removal in bioelectrochemical systems. Environ. Sci. Technol. 2011, 45 (24), 10557–66. 10.1021/es202047x. 22070656

[ref49] Liu S.; Yang F.; Xue Y.; Gong Z.; Chen H.; Wang T.; Su Z. Evaluation of oxygen adaptation and identification of functional bacteria composition for anammox consortium in non-woven biological rotating contactor. Bioresour. Technol. 2008, 99 (17), 8273–8279. 10.1016/j.biortech.2008.03.006. 18439820

