# Supplementary material for: Enhancement of Ammonium Oxidation at Microoxic Bioanodes
Source: Environ Sci Technol. 2023 Jul 27;57(31):11561–71. doi: 10.1021/acs.est.3c02227 (PMC10413939; doi:10.1021/acs.est.3c02227)
Supplement: Supplementary file 1 — es3c02227_si_001.pdf [file es3c02227_si_001.pdf]

Supporting information

Enhancement of ammonium oxidation at microoxic bioanodes

Xiaofang Yan [†], Dandan Liu [‡], Johannes B.M. Klok [§], Sanne M. de Smit [†], Cees J.N. Buisman[†,§], Annemiek ter Heijne [†]*

[†]Environmental Technology, Wageningen University & Research, P.O. Box 17, 6700 AA Wageningen, The Netherlands

[‡]Paqell B.V., Reactorweg 301, 3542 AD Utrecht, The Netherlands

[§]Wetsus, European Centre of Excellence for Sustainable Water Technology, Oostergoweg 9, 8911 MA Leeuwarden, The Netherlands

Summary

Page S3: Figure S1

Page S4: Figure S2

Page S5: Figure S3

Page S6: Figure S4

Page S7: Figure S5

Page S8-S9: Abiotic ammonium absorption experiments by GAC; Abiotic DO measurement of the analyte; N_2, H_2, N_2O, NH_2OH, NO_2^-, NO_3^- measurements; Electron balance calculation

Page S10: Calculation of thermodynamic potential of oxygen evolution

Page S11: Separate experiment for optimal ATU concentration determination

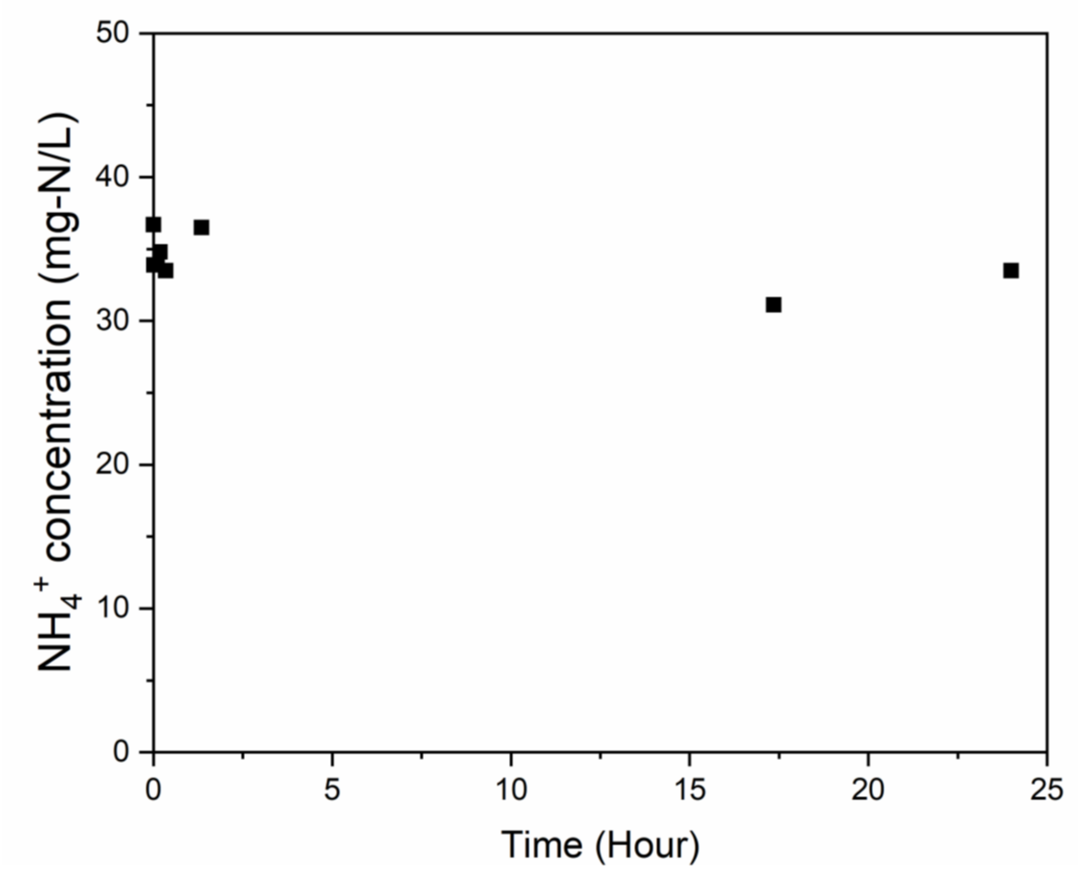

Figure S1. Variation of NH_4^+ concentration over a 24-hour period in a non-inoculated batch mode bioelectrochemical system. The data were collected from reactor 1.

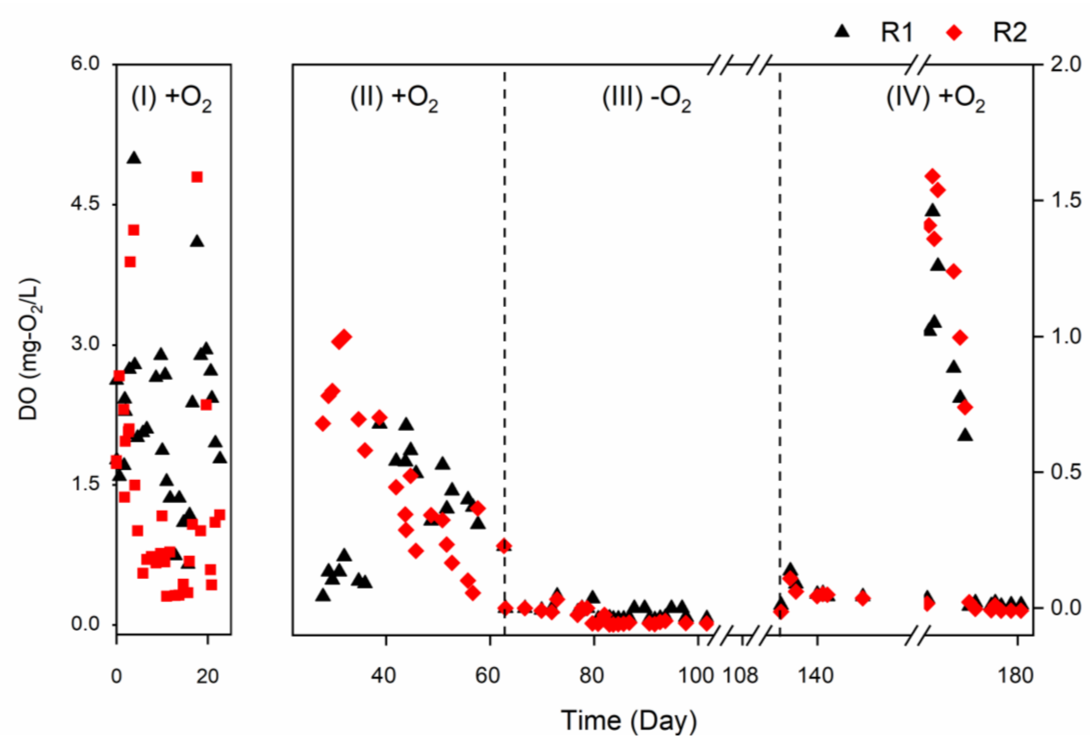

Figure S2. Time-dependent changes in dissolved oxygen levels within the anolyte during the experimental period. (I) Batch phase with oxygen (left Y-axis), (II) Continuous mode with oxygen, (III) Continuous phase without oxygen, (IV) Continuous phase with oxygen (right Y-axis). The breaks stand for the periods where reactors stabilized from switching between different conditions or disruption due to sampling. The data collected during these periods were not used in this study.

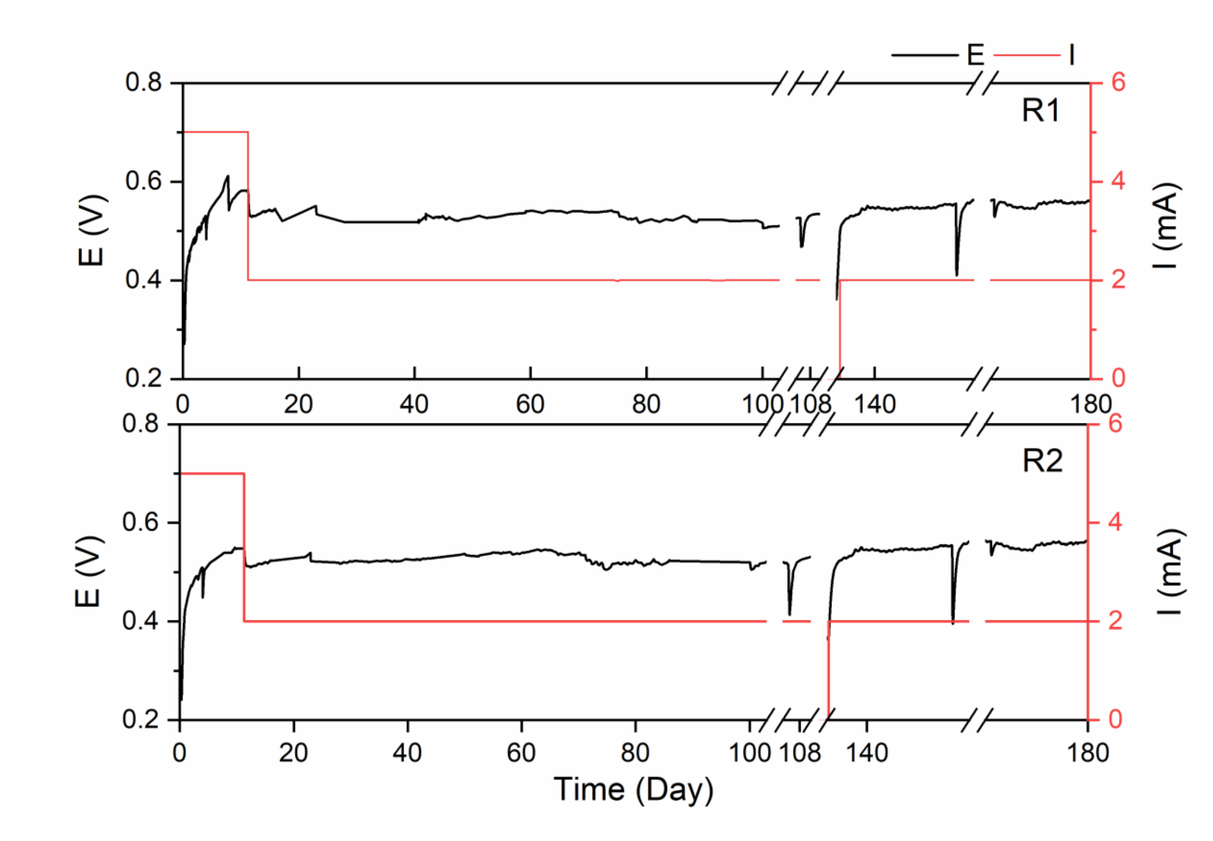

Figure S3. Evolutions of anode potentials. The BESs were continuously operated at 5 mA from day 0 to day 11 and afterwards were operated at 2 mA.

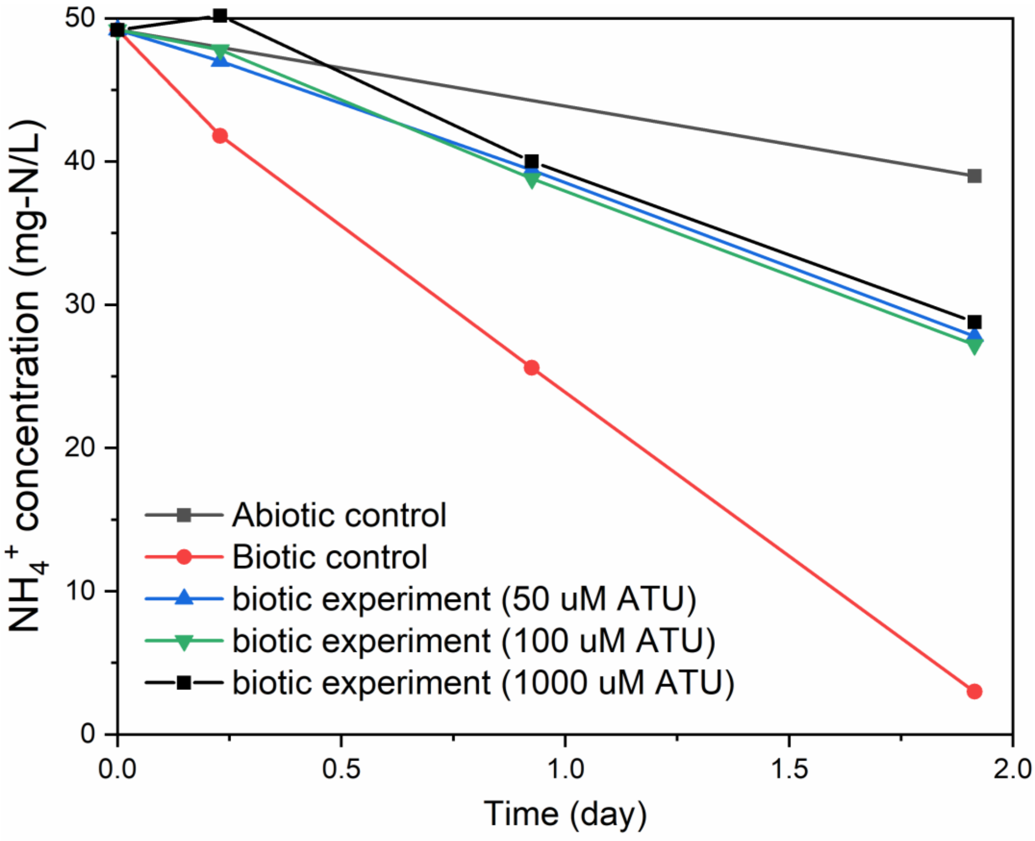

Figure S4. Ammonium concentration changes in batch experiments: comparison of abiotic control (without biomass or ATU), biotic control (without ATU but with biomass), and biotic experiments (with biomass and different concentrations of ATU).

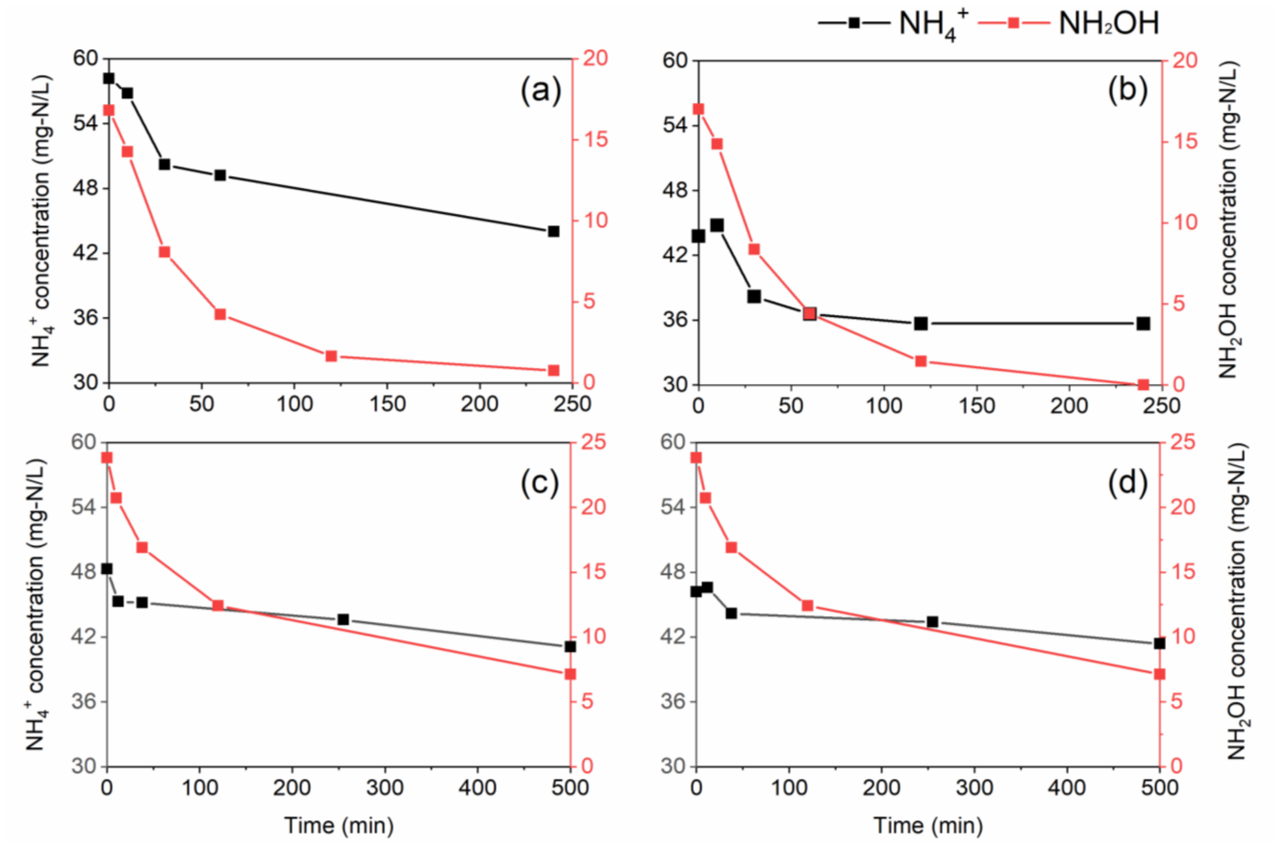

Figure S5. Variation of NH_4^+ and NH_2OH concentration during batch tests with NH_2OH and NH_4^+. (a) with oxygen R1; (b) with oxygen R2; (C) Without oxygen R1, (d) without oxygen R2.

Abiotic NH_4^+ absorption experiment by GAC

Before the inoculation of BESs, an abiotic experiment was conducted to assess the potential for GAC to absorb NH_4^+ in R1. This batch experiment was conducted in the presence of oxygen using a 500 mL ammonium solution (37 mg-N/L) prepared from the stock and medium solution. The solution was recirculated between the BES cell and recirculation bottle at the rate of 10 mL/min, while the recirculation bottle was stirred continuously. To monitor the NH_4^+ absorption by GAC, liquid samples were collected at 1 min, 5 min, 10 min, 20 min, 1.5 h, 17h, and 24h.

Abiotic DO measurement of the analyte

Before inoculating the BESs, an abiotic experiment was conducted to measure the DO levels of the analyte, specifically focusing on diffusion without oxygen consumption resulting from ammonium oxidation. The reactors were operated in continuous mode under open circuit potential. The experiment utilized only a medium solution without NH_4Cl as the influent, while the remaining operational parameters were consistent with the biotic microoxic experiment. The experiment concluded when the oxygen concentration of analyte reached a stable state, and the recorded oxygen concentration data was collected.

N_2, H_2, N_2O, NO_2^-, and NO_3^- measurements

NO_2^- and NO_3^- measurements were performed with an ion chromatography system (ICS-2100, Thermo Scientific Dionex, USA) using analytical column (IonPac AS17-C 2mm, Thermo Scientific Dionex, USA) and a conductivity detector. The column temperature was set at 30 °C. The injection volume was 10 µL and flow rate was 0.25 mL/min. The eluent was generated with milli-Q and KOH (5-30 mM). The N_2O concentration was measured with a gas chromatograph (Interscience Trace1300, Thermo Scientific, USA) equipped with the ECD detector and HAYESEP Q column (80-100 1/8" ss 3m, Agilent, USA). The column

temperature was 60 °C. The injection volume was 1 mL. N_2 and H_2 concentrations were analysed with a gas chromatograph (Hewlett-Packard 5890, Agilent, USA) equipped with TCD detector and HP Molsieve 5A column (30 m *0.53mm*25 µm). The column temperature was 40°C. The carrier gas was argon gas, and injection volume was 100 µL. NH_2OH was determined by Spectrophotometric based method. Briefly, 1 mL of the liquid samples were mixed with 1 mL PBS (0.05 M) and 0.8 mL water, 0.2 mL trichloroacetic acid (12% by weight), 1 mL 8-quinolinol solution (1% w/v) and 1 mL $NaHCO_3$ (1M), and heated at 100 °C for 1 min. After cooling down for 15 min, absorbance was measured at 705 nm.

Electron balance

The moles of electrons obtained were calculated using

$$n_{obtained} = \frac{\int_0^t I\,dt}{F}$$

Where I is the current (A) (I=0.05 A from day 0-11, and 0.02 A from day 12 onwards), dt (s) is the time interval over which data were collected. F is Faraday's constant (96485 C mol-1).

The moles of electrons donated was calculated using

$$n_{donated} = n_{N_2} * 6 + n_{N_2O} * 8 + n_{NO_2^-} * 6 + n_{NO_3^-} * 8$$

$n_{N_2}, n_{N_2O}, n_{NO_2^-}, n_{NO_3^-}$, is the mol of nitrogen products (N_2, N_2O, NO_2^-, NO_3^-) during the time interval over which data were collected. 6 is the number of electrons for producing 1 mol N_2 or NO_2^- from NH_4^+ oxidation. 8 is the number of electrons for 1 mol N_2O or NO_3^- from NH_4^+ oxidation.

The coulombic efficiency (CE) was calculated using

$$CE\ (\%) = \frac{n_{obtained}}{n_{donated}} * 100\%$$

Calculation of thermodynamic potential of oxygen evolution

We have calculated the thermodynamic potential for O_2/H_2O couple under our specific conditions (25 °C and pH 7.5, DO =0.02 mg/L), resulting in a value of + 0.50 V vs Ag/AgCl. The calculation processes are as follows:

Overall: $O_2 + 4H^+ + 4e^- \rightarrow 2H_2O$

$E=E_0$-RT/NF lnQ

E0=standard state cell potential E_0=+ 1.23 V (vs NHE)

R=constant (8.31 J/mol K)

T=298 K (measured value)

F=96485 C/mol

N=4 mols of electrons

pH=7.5 (measure value)

We calculate Q using molar concentrations for solutions, DO=0.02 mg/L=$6.25*10^{-7}$ mol/L

$$Q=\frac{1}{[H^+]^4 * P_{O_2}} = \frac{1}{[10^{-7.5}]^4 (6.25*10^{-7})^1} = 1.60 * 10\text{^}36$$

E=0.70 V VSSHE= 0.50 vs Ag/AgCl

Optimal ATU concentration determination

To determine the optimal ATU concentrations for effective inhibition, separate experiments were conducted using three different ATU concentrations (50, 100, 1000 uM). Nitrifier biofilms attached to a graphite felt originating from the dual reactor aerobic chamber were placed in a 500 mL beaker filled with the influent of the BES reactor. For the experimental groups, a predetermined amount of ATU solution was then added to the beaker to reach a desired concentration. The beakers were left open to the air and stirred using magnetic stirrers at the speed of 120 rpm/min for 48 h. The NH_4^+ and DO were measured. A biotic control group without ATU addition was included, along with an abiotic control conducted without biofilm and ATU addition. The inhibition performance were assessed by comparing the NH_4^+ consumption and DO between control groups and experimental groups.
